# Time Is Money: The Decision Making of Smartphone High Users in Gain and Loss Intertemporal Choice

**DOI:** 10.3389/fpsyg.2017.00363

**Published:** 2017-03-10

**Authors:** Zixuan Tang, Huijun Zhang, An Yan, Chen Qu

**Affiliations:** ^1^Center for Studies of Psychological Application, School of Psychology, South China Normal UniversityGuangzhou, China; ^2^Guangdong Key Laboratory of Mental Health and Cognitive Science, South China Normal UniversityGuangzhou, China; ^3^Scientific Laboratory of Economics Behaviors, School of Economics and Management, South China Normal UniversityGuangzhou, China

**Keywords:** smartphone high user, intertemporal choice, gain and loss, time perception, money perception

## Abstract

Nowadays the smartphone plays an important role in our lives. While it brings us convenience and efficiency, its overuse can cause problems. Although a great number of studies have demonstrated that people affected by substance abuse, pathological gambling, and internet addiction disorder have lower self-control than average, scarcely any study has investigated the decision making of smartphone high users by using a behavioral paradigm. The present study employed an intertemporal task, the Smartphone Addiction Inventory (SPAI) and the Barratt Impulsiveness Scale 11th version (BIS-11) to explore the decision control of smartphone high users in a sample of 125 college students. Participants were divided into three groups according to their SPAI scores. The upper third (69 or higher), middle third (from 61 to 68) and lower third (60 or lower) of scores were defined as high smartphone users, medium users and low users, respectively. We compared the percentage of small immediate reward/penalty choices in different conditions between the three groups. Relative to the low users group, high users and medium users were more inclined to request an immediate monetary reward. Moreover, for the two dimensions of time and money in intertemporal choice, high users and medium users showed a bias in intertemporal choice task among most of the time points and value magnitude compared to low users. These findings demonstrated that smartphone overuse was associated with problematic decision-making, a pattern similar to that seen in persons affected by a variety of addictions.

## Introduction

With the development and the popularization of mobile internet, most of us have our own mobile phone and can access the internet anytime and anywhere. Data from 2014 showed that mobile internet users accounted for 80% of internet users in China (Meeker, 2014). While the smartphone brings us convenience and efficiency, it can cause problems through overuse. Some researchers have even conceptualized severe overuse as a form of addiction. Shaffer (1996) defined an addiction behavior as one that: (1) brings pleasure and relieves pain and stress; (2) individuals cannot control even if it causes some harmful consequences. Actually many smartphone high users have reached these criteria, but smartphone overuse was not included in DSM-5 (Diagnostic and Statistical Manual of Mental Disorders, Fifth Edition) as an addiction. Therefore, the present study defined problematic use as “overuse,” similar to most other existing studies. And we define smartphone overuse as unwanted reliance on smartphones (Rush, 2011), and experiencing daily-life function disturbance by using smartphones over time (Lin et al., 2014).

Because smartphone is convenient, smartphone overuse is more extensive compared to internet addiction disorder, and does more harm. Up to December, 2011 in Korea, the National Information Society Agency (NIA) reported that smartphone addiction rates (8.7%) were higher than internet addiction rates (7.8%). Park and Lee (2014) also revealed that the diversity of applications and conveniences of smartphones could induce a higher addiction rate compared to the internet. Unlike internet addicts who indulge in online gaming (Yang and Tung, 2007), smartphone addicts were more likely to enjoy chatting, voice calls, or SNSs, and perceived smartphones more positively as fostering social relationships (Park and Lee, 2014). Smartphones also provide users with internet-based communication, business trading, education, entertainment media, and even clinical applications. Its increased importance in our daily lives perhaps explains how people can fail to control the impulse to use their smartphone, even if it is to do nothing more than unlock it (Lin et al., 2014).

There are similarities between smartphone overuse and other addictions. A great number of studies have reported that impulsivity is highly related to addiction (Businelle et al., 2010; Kishinevsky et al., 2012). For example, Dixon et al. (2006) used a Stroop paradigm to show that compared to healthy comparison subjects, pathological gamblers were more impulsive in terms of attention and response inhibition. Pathological gamblers, relative to the comparison subjects, also exhibited decreased activity in the left ventromedial prefrontal cortex, an area previously shown to be associated with impulse control (Potenza et al., 2014). Recently, a study showed that failure of self-regulation was likely related to a higher risk of addictive smartphone behavior (van Deursen et al., 2015), and smartphone high users had lower cognitive control (Chen et al., 2016). In the present study, we focus mainly on decision control of smartphone high users by using the intertemporal choice task, in which individuals need to control themselves to get a higher reward.

Intertemporal choice is defined as a decision that involves trade-offs among costs and benefits occurring at different times (Frederick et al., 2002). In most studies, individuals are required to choose between a small sooner (SS) reward and a large later (LL) reward. This is similar to “delay of gratification,” the delay of gratification is defined as the process of waiting for a LL reward and resisting temptation of the SS reward (Mischel, 1974). Both paradigms requires subjects to choose from two options, but they are rather different. The former emphasizes the decision making process, focusing on the higher cognitive process including calculation, analysis, logical reasoning, and trade-offs. While the latter emphasizes the execution process, focusing on the basic, instinctive response when subjects have made the choice to wait for the LL reward. That is, the emotion, willpower, and the motivation intensity in the waiting period. The delay of gratification task was mainly used in children, because adults had better self-control, thus the temptation of the reward in delay of gratification was not big enough for adults, and there were ceiling effect as the waiting time in lab was too short (Ren et al., 2015). Therefore, in the present study, we choose an intertemporal choice task to explore the decision control of smartphone high users.

According to economic theory, people should choose LL to maximize their earnings. However, Mazur (1984) proposed that money devalues subjectively over time such that people can prefer the immediately available reward, even if it is sometimes substantially smaller than the delayed option. This decision preference has been documented by some research (Kalenscher and Pennartz, 2008; Qu et al., 2013). Mazur also proposed a hyperbolic function to value the reward decreases over time: *SV = R/*(*1* + *kT*), where *SV* is the subjective value of the delayed reward *R* after a waiting time *T*, and *k* is the delay discount rate. Thus, the delay discounting rate *k* reflects the degree to which the subjective value decreases over time. With higher delay in discounting rate, the subjective value decreases more rapidly.

It has been found that behavior addicts (e.g., pathological gamblers or overeaters) have time discounting rates that are higher than control subjects (Bickel et al., 2012). Similar results have been found in internet addicts (Saville et al., 2011). However, it is still unknown whether smartphone overuse exerts an influence on time discounting rates in intertemporal choice. Thus the present study focused on decision-making in the form of intertemporal choice of smartphone high users. It is plausible that as people become more and more dependent on the convenience of smartphones, they also become generally less patient. The first major goal of this paper was to investigate whether the percentage of SS choices in an intertemporal task would be different between high smartphone users, medium users, and low users.

Intertemporal choice is affected by two dimensions: reward and time. Many researchers believe that performance on an intertemporal choice task is related to sensitivity to money. The hyperbolic model of intertemporal choice suggests the subjective value should decrease over time. This means people tend to devalue future rewards. However, Hariri et al. (2006) found that high discounters, in comparison to low discounters, exhibited greater overall ventral striatal activity in response to positive and negative feedback stimuli related to immediate rewards. This indicates that high discounters overvalue immediate rewards rather than undervalue future rewards. Another ERP study indicated similar results that immediate but not future rewards elicited the reward positivity, which is associated with reward processing. This supports the assumption that an overestimation of immediate reward generated the bias of SS in intertemporal choice (Cherniawsky and Holroyd, 2013).

The other view on intertemporal choice is that the preference of smaller sooner choices is related to sensitivity to time. High discounters choose more SS because of their amplified subjective time perception (Zauberman et al., 2009). According to the hyperbolic function: *SV = R/*(*1* + *kT*), the longer one perceives time, the less subjective value one feels, leading to a higher tendency to choose SS. Wittmann and Paulus (2008) suggested that differences time perception ability have an effect on the delay discounting rate. Overestimating time leads to more SS choices, while underestimating time leads to more LL choices. Thus the second major goal of this paper was to investigate smartphone high users’ sensitivity to time and money in intertemporal choice.

An interesting phenomenon in intertemporal choice is that people show different sensitivity when the outcome constitutes a gain or a loss, which is called a sign effect (Loewenstein, 1987). Numerous studies have indicated that the outcomes that constitute a gain are discounted at a higher rate than the outcomes that constitute a loss (Benzion et al., 1989; Shelley, 1994; Frederick et al., 2002), but there have been opposite conclusions drawn about these findings (Loewenstein, 1987; Thaler, 1991; Baker et al., 2003). The mainstream view of the sign effect suggests that there are different neural mechanisms associated with gain and loss (Gehring and Willoughby, 2002; Xu et al., 2009). Gehring and Willoughby (2002) found that a negative-polarity event-related brain potential, probably generated by a medial-frontal region in or near the anterior cingulate cortex, was greater in amplitude when a participant’s choice between two alternatives resulted in a loss than when it resulted in a gain. In another ERP research(Qu), manipulating immediate and delayed gain and loss in a gambling task, it was found a different pattern of gain condition and loss condition, the delay impacted feedback-related negativity (FRN) only in gain conditions, with delayed winning eliciting a more negative FRN than immediate winning, but no difference in loss conditions. Other researchers have provided evidence that the results in gain conditions could not be generalized to loss conditions (Xu et al., 2009). Thus the third major goal of this paper was to test the performance in intertemporal choice of smartphone high users in both gain and loss conditions.

To investigate our research questions, an intertemporal choice task and a series of measures were used. The reaction time and the percentage of SS choices in the intertemporal choice task were recorded and further calculated into the index of ΔAmount and ΔTime. Based on previous study results and our experimental design, we predicted high smartphone users in gain conditions would show a higher percentage of smaller sooner choices, faster responses, higher impulsivity traits, lower level of decision control, and higher tendency to make irrational decisions, but might be the opposite way in loss condition, resulting from different neural mechanisms of loss and gain. We also expected high users would show sharper differences between different ΔAmount and ΔTime conditions compared to low users.

## Materials and Methods

### Participants

This study was approved by the Human Research Ethics Committee of South China Normal University, and all participants provided written informed consent to participate. We conducted a power analysis using G^∗^Power 3.1.0 and found that for a 2-tailed *F*-test with a hypothesized effect size *f* = 0.29 [the same effect size in Chen et al. (2016)] and an ^∗^ = 0.05, at least 87 participants (29 participants in each group) were needed to achieve a power of 0.95. So we collected a sample of 125 students from South China Normal University was recruited to participate in this study (52 males, 73 females, mean age = 19.92, *SD* = 1.20). All of them were right handed, had no past or present major neurological injury or illness, and were paid 15 CNY (about 2.25 dollars) for their participation.

### Experimental Procedures

#### Intertemporal Choice Task

Participants were first instructed as to the nature of the task. The instructions were as follows:

Welcome to our experiment! In this experiment, you need to make a series of fictive monetary choices containing both gain and loss situations. There is no right or wrong answers, just choose the one you prefer. Pressing “F” represents you choose the left one while pressing “J” represents the right one. The choices you made will not influence the payment of your participation, but please think it over to make every choice.

**FIGURE 1:**
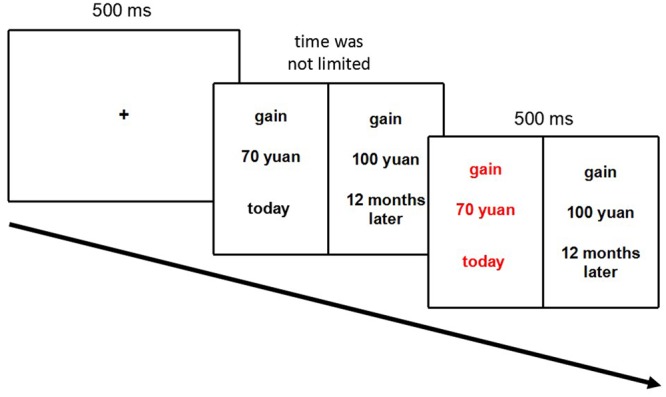
**Procedure of the intertemporal choice task.** Participants were required to choose the option that they preferred. The one they chose would subsequently turn red as feedback. In this trial, the ΔTime was 12 months and the ΔAmount was 30 yuan. In the experiment, the task was presented in Chinese.

After participants made a choice, the chosen option would turn red as feedback (see **Figure 1** for details). The experiment was presented in E-Prime 2.0. Given previous evidence suggesting no difference between the devaluation of real and fictive outcomes in delay-discounting studies, we did not deem it necessary for participants to actually receive money corresponding to their choices (Bickel et al., 2009).

The computerized task consisted of 146 trials, including 2 trials for practice and 144 experimental trials. The 144 experimental trials were divided into 2 blocks of 72 choices. One block is gain condition and another is loss condition, the two blocks were counter-balanced. Within each block the following variables were manipulated: waiting time for SS options (today, 3, 6, and 9 months), waiting time for LL options (3, 6, 9, and 12 months), reward/penalty values for SS options [10 yuan (about 1.45 dollars), 20 yuan, 30 yuan, 40 yuan, 50 yuan, 60 yuan, 70 yuan, 80 yuan, 90 yuan (about 13.08 dollars)], and reward/penalty value for LL options (100 yuan).

Inside each block, every amount from 10 to 90 yuan was repeated eight times (four times on the left side of the screen and four times on the right side), and the 100 yuan offer was repeated 36 times (18 times per side). Therefore, the value magnitudes could be divided into nine magnitude categories, and the frequency of each ΔAmount (the difference between every combination of LL and SS presented during the task) was equal. In order to keep the frequency of each ΔTime (the difference between waiting time associated with the LL and SS options) equal, the intertemporal characteristics of the experimental choices were divided into eight delay categories. We represent each delay category as a pair of alternatives, D vs. D′, where D is the delay to the early reward/penalty and D′ is the delay to the alternative later reward/penalty. The eight delay categories are as follows: today vs. 12 months, 12 months vs. today, today vs. 9 months, 12 months vs. 3 months, 9 months vs. 3 months, 6 months vs. 12 months, 9 months vs. 6 months, and 3 months vs. 6 months. In total, there were 72 pairs of choices for every block, and pairs of stimuli were displayed randomly (see **Table 1**). The purpose of keeping each ΔTime and ΔAmount equal was to better understand how both the amount of reward/penalty and the delay time influenced decisions.

**Table 1:** Pairs of stimuli.

ΔAmount ΔTime	ΔTime = 3 months	ΔTime = 6 months	ΔTime = 9 months	ΔTime = 12 months
ΔAmount = 10 yuan	**SS**: 3 (or 6) M, ¥90; **LL**: 6 (or 9) M, ¥100.	**SS**: 3 (or 6) M, ¥90; **LL**: 9 (or 12) M, ¥100.	**SS**: today (or 3 M), ¥90; **LL**: 9 (or 12) M, ¥100.	**SS**: today, ¥90; **LL**: 12 M, ¥100.
ΔAmount = 20 yuan	**SS**: 3 (or 6) M, ¥80; **LL**: 6 (or 9) M, ¥100.	**SS**: 3 (or 6) M, ¥80; **LL**: 9 (or 12) M, ¥100.	**SS**: today (or 3 M), ¥80; **LL**: 9 (or 12) M, ¥100.	**SS**: today, ¥80; **LL**: 12 M, ¥100.
ΔAmount = 30 yuan	**SS**: 3 (or 6) M, ¥70; **LL**: 6 (or 9) M, ¥100.	**SS**: 3 (or 6) M, ¥70; **LL**: 9 (or 12) M, ¥100.	**SS**: today (or 3 M), ¥70; **LL**: 9 (or 12) M, ¥100.	**SS**: today, ¥70; **LL**: 12 M, ¥100.
ΔAmount = 40 yuan	**SS**: 3 (or 6) M, ¥60; **LL**: 6 (or 9) M, ¥100.	**SS**: 3 (or 6) M, ¥60; **LL**: 9 (or 12) M, ¥100.	**SS**: today (or 3 M), ¥60; **LL**: 9 (or 12) M, ¥100.	**SS**: today, ¥60; **LL**: 12 M, ¥100.
ΔAmount = 50 yuan	**SS**: 3 (or 6) M, ¥50; **LL**: 6 (or 9) M, ¥100.	**SS**: 3 (or 6) M, ¥50; **LL**: 9 (or 12) M, ¥100.	**SS**: today (or 3 M), ¥50; **LL**: 9 (or 12) M, ¥100.	**SS**: today, ¥50; **LL**: 12 M, ¥100.
ΔAmount = 60 yuan	**SS**: 3 (or 6) M, ¥40; **LL**: 6 (or 9) M, ¥100.	**SS**: 3 (or 6) M, ¥40; **LL**: 9 (or 12) M, ¥100.	**SS**: today (or 3 M), ¥40; **LL**: 9 (or 12) M, ¥100.	**SS**: today, ¥40; **LL**: 12 M, ¥100.
ΔAmount = 70 yuan	**SS**: 3 (or 6) M, ¥30; **LL**: 6 (or 9) M, ¥100.	**SS**: 3 (or 6) M, ¥30; **LL**: 9 (or 12) M, ¥100.	**SS**: today (or 3 M), ¥30; **LL**: 9 (or 12) M, ¥100.	**SS**: today, ¥30; **LL**: 12 M, ¥100.
ΔAmount = 80 yuan	**SS**: 3 (or 6) M, ¥20; **LL**: 6 (or 9) M, ¥100.	**SS**: 3 (or 6) M, ¥20; **LL**: 9 (or 12) M, ¥100.	**SS**: today (or 3 M), ¥20; **LL**: 9 (or 12) M, ¥100.	**SS**: today, ¥20; **LL**: 12 M, ¥100.
ΔAmount = 90 yuan	**SS**: 3 (or 6) M, ¥10; **LL**: 6 (or 9) M, ¥100.	**SS**: 3 (or 6) M, ¥10; **LL**: 9 (or 12) M, ¥100.	**SS**: today (or 3 M), ¥10; **LL**: 9 (or 12) M, ¥100.	**SS**: today, ¥10; **LL**: 12 M, ¥100.

*Inside each block, there were four different ΔTime and nine different ΔAmount. Each ΔTime consisted of two different delay categories except when the ΔTime was 12 months. To keep each ΔTime equal, options in ΔTime = 12 months were repeated. “3 (or 6) M” means “3 (or 6) months” and “¥100” means “100 yuan.”*

#### Measures

Participants’ smartphone usage was assessed using the Smartphone Addiction Inventory (SPAI; Lin et al., 2014). There were 26 items divided into four subscales: Compulsive Behavior (such as “I feel distressed or down once I cease using smartphone for a certain period of time”), Withdrawal (such as “The idea of using smartphone comes as the first thought on mind when waking up each morning”), Tolerance (such as “I was told more than once that I spent too much time on smartphone.”), and Functional Impairment (such as “I feel aches and soreness in the back or eye discomforts due to excessive smartphone use”). The participants rated items on a 4-point Likert scale (1 = “strongly disagree,” 4 = “strongly agree”). So that the SPAI total scores ranged from 26 to 104. The Cronbach’s alpha coefficient for the SPAI is 0.94 and the Cronbach’s alpha coefficient in our sample is 0.90. For the four subscales, the Cronbach’s alpha coefficient in our sample is 0.69 (for Compulsive Behavior), 0.76 (for Withdrawal), 0.56 (for Tolerance), and 0.79 (for Functional Impairment).

General impulsivity was assessed using the Barratt Impulsiveness Scale 11th version (BIS-11; Patton et al., 1995) in Chinese. There were 30 items divided into three subscales: Attentional Impulsiveness (such as “I am restless at the theater or lectures”), Motor Impulsiveness (such as “I buy things on impulse”), Non-planning Impulsiveness (such as “I get easily bored when solving thought problems”). The participants were asked to rate items on a 4-point Likert scale (1 = “never,” 4 = “always”). The Cronbach’s alpha coefficient for the BIS-11 is 0.80 for Chinese adolescents (Yang, 2007), and the Cronbach’s alpha coefficient in our sample was 0.76. For the three subscales, the Cronbach’s alpha coefficient in our sample is 0.67 (for Attentional Impulsiveness), 0.55 (for Motor Impulsiveness), and 0.65 (for Non-planning Impulsiveness). And the validity of this scale was shown to be good by many researchers (Zhou, 2006; Yang, 2007; Yao et al., 2007).

### Statistical Analysis

We aimed to quantify the decision making in intertemporal choice by estimating the percentage of SS choices in each valence (gain or loss), ΔTime, and ΔAmount. Firstly we conducted correlation analysis to explore the relationship between the usage of smartphone and the percentage of SS choices. Then more detailed analyses on differences between high smartphone users, medium users, and low users were performed using several mixed ANOVA for each valence, and for both ΔTime and ΔAmount.

Meanwhile, we divided the participants into three groups by their total scores on the SPAI. Each group was classified according to use: the upper third of SPAI scores (69 or higher, *n* = 42) as the high users group; the middle third of SPAI scores (from 61 to 68, *n* = 42) as the medium users group; and the lower third of SPAI scores (60 or lower, *n* = 41) as the low users group. There were no differences between the three groups in gender [high users: 12 males, 30 females; medium users: 19 males, 23 females; low users: 21 males, 20 females; χ^2^(2, *N* = 125) = 3.175, *p* = 0.094], age [high users: *M* = 19.79, *SD* = 1.07; medium users: *M* = 19.86, *SD* = 1.26; low users: *M* = 20.12, *SD* = 1.27; *F*(2,122) = 0.90, *p* = 0.411], and monthly consumption [high users: *M* = 1170.37, *SD* = 411.29; medium users: *M* = 1104.00, *SD* = 272.31; low users: *M* = 1072.73, *SD* = 435.27; *F*(2,71) = 0.36, *p* = 0.699]. Results were also examined in relation to reaction time, delay discounting rate and the Barratt Impulsiveness Scale.

Analyses were performed using SPSS17.0, the significance level was set at *p* = 0.05. *T*-test, χ^2^ test, one way ANOVA, Repeated Measures ANOVA (RM-ANOVA; with Greenhouse-Geisser adjusted *p*-values) and Pearson correlation analyses were applied to analyze behavioral outcomes of performance on the intertemporal choice task. Simple effects were explored and interaction sources were systematically examined.

## Results

### Assessment of Demographic Variables

In order to test the effects of demographic variables, we recorded the gender, age, and socio-economic background of participants. The socio-economic background was quantified by the monthly financial consumption of each participant. Due to privacy issues, only 74 students provided their monthly consumption producing an average of 1118.92 (*SD* = 411.29) CNY (about US$167.91).

A *T*-test revealed that although female participants had a higher score on SPAI than male participants [female: *M* = 66.28, *SD* = 10.73; male: *M* = 61.62, *SD* = 10.58; *t*(123) = 2.41, *p* = 0.017], they showed no difference on intertemporal choice task performance, neither in gain [*F*(1,123) = 0.006, *p* = 0.936, $\eta_{p}^{2}$ < 0.001] nor loss condition [*F*(1,123) = 0.001, *p* = 0.981, $\eta_{p}^{2}$ < 0.001]. The correlation between age and SPAI score was significant (*r* = -0.178, *p* = 0.047), but the correlation between age and intertemporal choice task decision making were not significant neither in gain (*r* = -0.062, *p* = 0.495) nor loss condition (*r* = 0.088, *p* = 0.330). When the same analyses were performed on monthly consumption we found no significant correlations between it and SPAI score (*r* = 0.093, *p* = 0.429), gain condition (*r* = -0.042, *p* = 0.724), and loss condition (*r* = -0.034, *p* = 0.772). Since these factors (gender, age, and socio-economic background) had no effect on the decision making of intertemporal choice task, we did not include them in the following analyses.

### Assessment of Smartphone Usage and Impulsivity

Firstly we conducted a Pearson correlation on SPAI and BIS scores, including all of the three subscales. The results suggested that SPAI and BIS scores were positively related (*r* = 0.223, *p* = 0.012), revealing that the more frequently one uses their smartphone, the higher their level of impulsivity. A significant correlation was also found for the Attentional Impulsiveness subscale (*r* = 0.361, *p* < 0.001), but not for Motor Impulsiveness (*r* = -0.015, *p* = 0.871) nor Non-planning Impulsiveness (*r* = 0.151, *p* = 0.093) subscales.

Importantly, the three groups differed in Attentional Impulsiveness subscale of the BIS and the total BIS score (see **Table 2** for details). Multiple comparisons revealed that differences between each group were significant on the Attentional Impulsiveness subscale (all *p* < 0.001). For the Non-planning Impulsiveness subscale, the difference between high users and low users was significant (*p* = 0.035). For the total BIS score, the differences between high users and low users and the differences between medium users and low users were significant (*p* = 0.017 and *p* < 0.001, respectively). Measuring a decreasing attentional impulsiveness from low users to high users. High users and medium users also showed a relative impulsivity and a deficit in planning their behavior. These results indicated that high smartphone users and medium users had a higher trait level of general impulsivity.

**Table 2:** Results of impulsivity.

Subscale	High users		Medium users		Low users		Difference			
	*M*	*SD*	*M*	*SD*	*M*	*SD*	*F*	Significance	$\eta_{p}^{2}$	Power
BIS – AI	19.71	2.91	18.14	2.75	16.51	2.61	13.98	<0.001	0.186	0.998
BIS – MI	22.36	3.71	22.48	3.36	21.24	3.46	1.55	0.217	0.025	0.323
BIS – NI	27.69	4.49	26.95	3.4	25.81	4.08	2.31	0.104	0.036	0.460
BIS – Total	69.76	8.00	67.57	6.84	63.56	7.70	7.22	0.001	0.106	0.930

*According to the scoring guide, we assessed the trait level of general impulsivity for both groups. BIS – AI, BIS – MI, and BIS – NI refers to the subscale of Attentional Impulsiveness, Motor Impulsiveness, and Non-planning Impulsiveness, respectively.*

### Behavioral Performance of Intertemporal Choice

#### Reaction Times

We conducted a one-way ANOVA on reaction times (RTs, ms) using smartphone usage (high users/medium uses/low users) as the independent variable. The difference between the three groups was not significant [high users: *M* = 2733.91, *SD* = 1205.42; medium users: *M* = 2656.40, *SD* = 1209.39; low users: *M* = 2231.69, *SD* = 1018.35; *F*(2,122) = 2.29, *p* = 0.106, $\eta_{p}^{2}$ = 0.036, *power* = 0.458].

#### Percentage of SS Choices

Firstly, we conducted a Pearson correlation on the SPAI score and the percentage of SS choices in gain and loss conditions. We found no significant correlations for either condition (gain: *r* = 0.133, *p* = 0.139; loss: *r* = -0.140, *p* = 0.118). However, these non-significant correlations can be explained by the non-linearity seen next.

**FIGURE 2:**
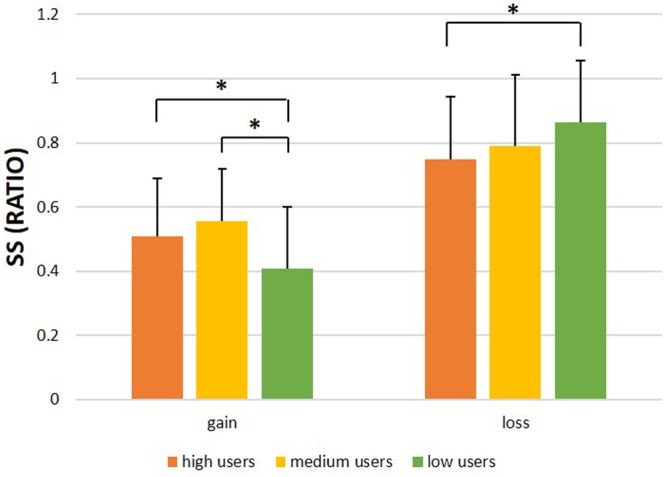
**Performance results.** The percentage of smaller sooner (SS) choices statistically differed across the high smartphone users groups, the medium users group, and the low users group. With high smartphone users and medium users showing a higher percentage of SS than low users in the gain condition and high smartphone users showing a lower percentage of SS than low users in the loss condition. ^∗^Significant difference refers to *p* < 0.05. Error bars show one standard error.

To examine the differences in choices, we compared the percentage of SS option choices between the three groups. In the gain condition, there was a significant difference between the three groups, *F*(2,122) = 6.76, *p* = 0.002, $\eta_{p}^{2}$ = 0.100, *power* = 0.912. High users selected 50.89% of SS in average (*SD* = 0.18), medium users selected 55.45% of SS in average (*SD* = 0.16), whereas low users selected only 40.71% (*SD* = 0.21). Multiple comparisons suggested that the difference between low users and medium users (*p* < 0.001) and the difference between low users and high users (*p* = 0.014) was significant. In the loss condition, there was a significant difference between the three groups, *F*(2,122) = 3.335, *p* = 0.039, $\eta_{p}^{2}$ = 0.052, *power* = 0.621. High users selected 74.83% of SS in average (*SD* = 0.20), medium users selected 79.06% of SS in average (*SD* = 0.22), whereas low users selected 86.26% (*SD* = 0.19) (see **Figure 2**). Multiple comparisons suggested that only the difference between low users and high users (*p* = 0.012) was significant. However, as the observed statistical power was lower than the standards for these analyses, these results should be interpreted with caution. These results indicated that in the gain condition, high smartphone users and medium users tended to choose the immediately available reward more often. In the loss condition, high users were tended to take the penalty later more often; however, there was no difference between high users and medium users both in gain and loss conditions.

**FIGURE 3:**
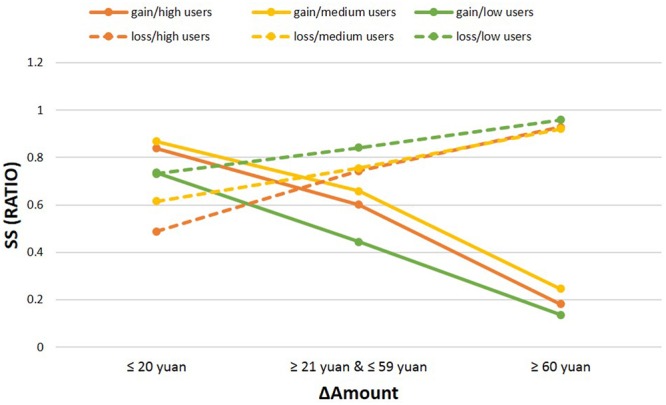
**Performance results for different ΔAmount.** Sooner Small (SS) choices are presented separately according to increasing difference in the magnitude of rewards/penalties. In the gain condition, significant differences are displayed between medium users and low users in all ranges, and high smartphone users and low users in the small and medium range. In the loss condition, the significant difference between high smartphone users and low users appeared only in the small range.

As hypothesized, we calculated two indices that allowed us to better understand how both the amount of reward/penalty and the delay time influenced decisions between the three groups. One possibility is that high smartphone users and medium users differ from low users in the way that they weigh the amount of reward or the amount of waiting time to obtain a reward. Therefore, we calculated the ΔAmount and the ΔTime. First we separated the percentage of SS choices according to three different ΔAmount values, grouped in the following way: LL – SS = 20 yuan or less (small Δ), from 21 to 59 yuan (medium Δ), 60 yuan or above (large Δ). The justification of the way to combine ΔAmount was tested (please see Supporting Information “The Test between Different ΔAmount” for details). A mixed ANOVA was conducted using percentage of SS choices as the dependent variable, ΔAmount (Δ ≤ 20, 30 ≤ Δ ≤ 60, Δ ≥ 70) and valence (gain vs. loss) as within-subjects factors, and group (high users/medium users/low users) as the between-subjects factor. The main effect of group was not significant [*F*(2,122) = 1.59, *p* = 0.209, $\eta_{p}^{2}$ = 0.025, *power* = 0.331]. The main effect of valence was significant [*F*(1,122) = 11.94, *p* < 0.001, $\eta_{p}^{2}$ = 0.385, *power* = 1.000]. Participants chose a significantly higher percentage of SS options in the loss condition compared to the gain condition. The main effect of ΔAmount was significant [*F*(2,244) = 73.46, *p* < 0.001, $\eta_{p}^{2}$ = 0.376, *power* = 1.000]. Multiple comparisons showed that in the loss condition, participants chose a significantly lower percentage of SS options for a small ΔAmount than for medium (*p* < 0.001) and large ΔAmount (*p* < 0.001), and participants chose a significantly lower percentage of SS options for medium ΔAmount than for large ΔAmount (*p* < 0.001); but in gain condition, participants chose a significantly higher percentage of SS options for a small ΔAmount than for medium (*p* < 0.001) and large ΔAmount (*p* < 0.001), and participants chose a significantly higher percentage of SS options for medium ΔAmount than for large ΔAmount (*p* < 0.001). Moreover, the interaction of the three factors was significant [*F*(4,244) = 3.45, *p* = 0.012, $\eta_{p}^{2}$ = 0.054, *power* = 0.819]. Multiple comparisons showed that in the loss condition, only when it is small ΔAmount, high users chose significantly fewer SS options than low users (*p* = 0.002). In the gain condition, medium users chose significantly more SS options than low users in all ΔAmount (*p* = 0.012 for small ΔAmount, *p* < 0.001 for medium ΔAmount, *p* = 0.002 for large ΔAmount), and high users chose significantly more SS options than low users with a small (*p* = 0.050) and medium (*p* = 0.008) ΔAmount. However, high users and medium users did not differ in all ΔAmount (see **Table 3** and **Figure 3** for details).

**Table 3:** Percentage of SS choices between groups in different ΔAmount.

ΔAmount		High users		Medium users		Low users		Difference			
		*M*	*SD*	*M*	*SD*	*M*	*SD*	*F*	Significance	$\eta_{p}^{2}$	Power
Gain	≤20 yuan	0.84	0.21	0.87	0.21	0.74	0.28	3.60	0.030	0.056	0.657
	≥21 yuan and ≤59 yuan	0.60	0.27	0.66	0.21	0.44	0.31	7.26	0.001	0.106	0.931
	≥60 yuan	0.18	0.16	0.25	0.16	0.14	0.16	4.95	0.009	0.075	0.801
Loss	≤20 yuan	0.49	0.35	0.62	0.35	0.73	0.35	5.10	0.007	0.077	0.813
	≥21 yuan and ≤59 yuan	0.74	0.28	0.75	0.28	0.84	0.26	1.58	0.211	0.025	0.329
	≥60 yuan	0.93	0.11	0.92	0.16	0.96	0.09	1.08	0.343	0.017	0.236

**FIGURE 4:**
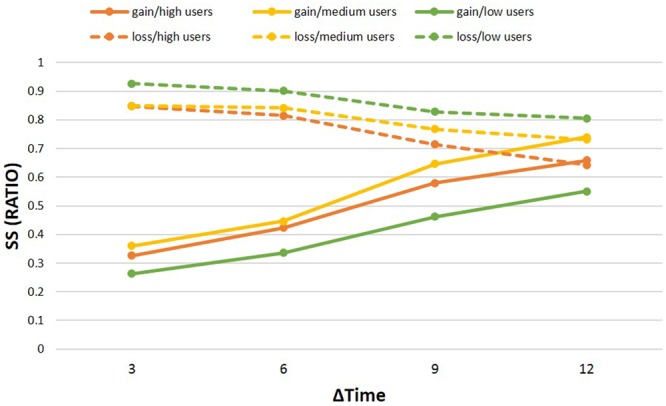
**Performance results for different ΔTime.** Sooner small (SS) choices are shown for the 4 months intervals. In the loss condition, the high users and low users differed in all ΔTime, and medium users differed from normal when Δ = 3, high users and medium users did not differ in all ΔTime; in the gain condition, medium users differed in all ΔTime, and high users differed from low users when Δ = 6 and Δ = 9, but high users and medium users did not differ in all ΔTime.

In the second calculation, which involved the factor waiting time, we calculated ΔTime and split the number of SS choices into the four intervals of delay used in the experimental task: 3, 6, 9, and 12 months of difference between the SS and LL alternatives. The justification of the way to combine delay categories was tested (please see Supporting Information “The Test between Different Delay Categories” for details). We also explored the differences between high users and low users in the four specific time points. First, a mixed ANOVA was conducted using the percentage of SS choices as the dependent variable, ΔTime (3, 6, 9, and 12 months) and valence (gain vs. loss) as within-subjects factors, and group (high users/medium users/low users) as the between-subjects factor. The main effect of group was not significant [*F*(2,122) = 1.72, *p* = 0.184, $\eta_{p}^{2}$ = 0.027, *power* = 0.354], whereas the main effect of valence was significant [*F*(1,122) = 146.02, *p* < 0.001, $\eta_{p}^{2}$ = 0.545, *power* = 1.000]. Participants chose a significantly higher percentage of SS options in the loss condition compared to the gain condition. The main effect of ΔTime was also significant [*F*(3,366) = 39.99, *p* < 0.001, $\eta_{p}^{2}$ = 0.247, *power* = 1.000]. Participants chose a higher percentage of SS choices with higher ΔTime in gain condition and contrary in loss condition (all *p* < 0.005). The interaction between the three factors was not significant [*F*(6,366) = 1.611, *p* = 0.176, $\eta_{p}^{2}$ = 0.026, *power* = 0.476]. In order to explore the detailed effects at specific time points, we conducted multiple comparisons of the specific time points in loss and in gain condition, respectively. In the loss condition, the high users chose significantly less SS options than low users in all ΔTime (*p* = 0.028 for Δ = 3, *p* = 0.037 for Δ = 6, *p* = 0.030 for Δ = 9, *p* = 0.007 for Δ = 12), medium users chose significantly fewer SS options than low users when Δ = 3 (*p* = 0.030), but high users and medium users did not differ in each ΔTime. In the gain condition, medium users chose significantly more SS options than low users in all ΔTime (*p* = 0.008 for Δ = 3, *p* = 0.013 for Δ = 6, *p* < 0.001 for Δ = 9, *p* = 0.001 for Δ = 12), high users chose significantly more SS options than low users when Δ = 6 (*p* = 0.013) and when Δ = 9 (*p* = 0.050), but high users and medium users did not differ in each ΔTime (see **Table 4** and **Figure 4** for details).

**Table 4:** Percentage of SS choices between groups in different ΔTime.

ΔTime		High users		Medium users		Low users		Difference			
		*M*	*SD*	*M*	*SD*	*M*	*SD*	*F*	Significance	$\eta_{p}^{2}$	Power
Gain	3	0.33	0.16	0.36	0.13	0.26	0.19	3.70	0.028	0.057	0.670
	6	0.42	0.20	0.45	0.19	0.34	0.21	3.49	0.033	0.054	0.643
	9	0.58	0.21	0.65	0.21	0.46	0.25	7.08	0.001	0.104	0.925
	12	0.66	0.22	0.74	0.24	0.55	0.28	6.07	0.003	0.091	0.879
Loss	3	0.85	0.16	0.85	0.20	0.93	0.12	3.23	0.043	0.050	0.607
	6	0.81	0.18	0.84	0.20	0.90	0.17	2.34	0.101	0.037	0.466
	9	0.71	0.23	0.77	0.24	0.83	0.24	2.42	0.094	0.038	0.479
	12	0.64	0.28	0.73	0.27	0.81	0.26	3.75	0.026	0.058	0.676

#### Delay Discounting Rate (*k*)

To examine how participants discounted rewards/penalties according to waiting time, we calculated the discount factor. The discount rate can be described with a hyperbolic function as follows: *SV* = *R/*(*1* + *kT*). In the present study, the discount rate can be calculated by comparing an “SS” option with an “LL,” when the “SS” is chosen, with the following formula:

(1) $$\begin{array}{c} k=(RLL-RSS)/({\mathrm{RSS}}^{*}{TLL-RSS}^{*}\mathrm{TSS}) \end{array}$$

Where “RLL” is the reward/penalty linked to the “LL” option, “RSS” is the reward/penalty linked to the “SS” option, “TLL” is the waiting time associated with the “LL” option, and “TSS” is the waiting time associated with the “SS” option. The final *k*-value was computed per participant as a mean of the *k*-values calculated for every option that participants selected.

We conducted a mixed ANOVA with the *k*-value as the dependent variable, valence (gain vs. loss) as the within-subjects factor, and group (high users/medium users/low users) as the between-subjects factor. The effect of group was found not to be significant [*F*(2,122) = 2.20, *p* = 0.116, $\eta_{p}^{2}$ = 0.035, *power* = 0.441]. However, the effect of valence was significant [*F*(1,122) = 919.50, *p* < 0.001, $\eta_{p}^{2}$ = 0.883, *power* = 1.000]. Participants had a higher discounting rate in the loss condition than in the gain condition. The interaction was not significant [*F*(2,122) = 2.45, *p* = 0.090, $\eta_{p}^{2}$ = 0.039, *power* = 0.485]. Multiple comparisons suggested that the only significant result appeared between high users and low users in the loss condition (mean of high users’ *k* = 0.44, mean of low users’ *k* = 0.50, *p* = 0.030).

Finally, as we can see, both smartphone use and impulsiveness have an effect on the behavior in intertemporal choice task. Thus, we did several similar analyses using the BIS score as a covariate. To explore whether the different performance between groups were due to smartphone overuse or trait impulsivity. The results showed that the BIS score did not have a significant effect on intertemporal choice (please see Supporting Information “BIS Score as A Covariate” for details).

## Discussion

The present study explored the decision making of smartphone high users in intertemporal choices concerning gain and loss. Here are our major findings: (a) high smartphone users and medium users were more impulsive than low users; (b) With an intertemporal task, comparing to low users, it was validated that high smartphone users and medium users have a higher tendency to make irrational decisions, no significant differences between high smartphone users and medium users in all conditions of the intertemporal task; (c) the discounting rate in the gain condition was much lower than in the loss condition for all the three groups; (d) the results supported our hypotheses and are important because we separate time and money, and thus showed that high smartphone users and medium users showed a bias in intertemporal choice task among most of the time points and value magnitude compared to low users.

An interesting phenomenon in the present study is that the behavioral pattern of high smartphone users and medium users in intertemporal choice was almost the same. Indeed, medium smartphone users even had a higher percentage of SS choices in the gain condition. This result suggests that after a certain critical amount of smartphone usage, the influence on intertemporal choice does not increase as a function of smartphone usage. In the present study, the critical score of smartphone usage is 61, this is consistent with the results of Chen et al. (2016), in which they define 62.46 or higher of SPAI scores as the excessive smartphone use group, and the samples in their study were also students.

The analyses of SS choices revealed that compared to the low users group, high and medium users tended to choose SS options in the gain condition but LL options in the loss condition, which reflected their preference for an immediately available reward and a later penalty. Consistent with previous studies, these results were consistent with our hypothesis that smartphone high users showed a distinct decision-making bias toward immediate rewards and later penalties compared to low users. In addition to a paper-based delay-discounting task, Saville et al. (2011) conducted a survey in order to measure individual levels of internet addiction, their results indicated that Internet addicts discounted delayed rewards faster than non-Internet addicts. Xu (2012) employed a Chinese version of the computerized Iowa Gambling Task to examine internet addicts’ decision making functions. These findings indicated that Internet addicts have deficits in decision making functions, which are characterized by an immediate win-priority selection pattern and tolerance to high risk. The present study showed that smartphone high users preferred immediate reward, suggesting that smartphone overuse exhibited dysfunctional decision-making as one sign of behavioral addiction.

We propose that in this smartphone era, humans enjoy the “immediate rewards” brought about by the convenience and efficiency of smartphones, such as instant information and social connection, like never before. Sheldon et al. (2011) found that feelings of unhappiness and disconnection with others drove people’s use of Facebook, because using Facebook made them feel re-connected and this feeling reinforced the use of Facebook. A review by Wilson et al. (2012) pointed out that the most common internal motivation to use Facebook was users’ desire to keep in touch with friends, which could expand to “social capital” – the benefits received from relationships with other people. In addition, the smartphone allows us to maintain our interpersonal relationships in a convenient and efficient way (Gosling, 2009). Thus, we could speculate that smartphone high users might be more impatient because they are more used to “immediate reward.”

To further understand how reward/penalty amount and time delay influenced participants’ choices, we computed the ratio of SS choices for three intervals of value magnitude and four intervals of time delay magnitude. We found that, in the loss condition, all participants behaved the same way when the difference in terms of amount between the options was large (more than 60 yuan). That is, high users selected the smaller sooner option more than 90%. When the difference in magnitude decreased (medium values), the difference between high users and low users becomes larger, but still not significant. For the smallest difference in amount, while low users were still biased toward the SS option, smartphone high users showed no preference between SS and LL (indifference point, 48%). This implies that smartphone high users were more sensitive to the changes of value magnitude than low users. This result was similar to that found in research by Grecucci et al. (2014). The same analysis applied to delay revealed that for the loss condition, larger differences in delays led high users to select less SS options as compared with low users. While in the gain condition, relatively smaller differences in delays led high users to select more SS options as compared with low users. Thus, our results suggest that smartphone high users might differ from low users, both in the way that they weight the amount of reward and the amount of time that they are willing to wait to obtain a reward.

Finally, we explored the decision making of high users from the perspective of sign effect (gain or loss). Consistent with previous studies, the discounting rate in the loss condition was much higher than in the gain condition (Benzion et al., 1989; Shelley, 1994; Frederick et al., 2002), and the decision making of smartphone high users was different in gain condition and loss condition. These findings are interesting in light of a neuroimaging study that revealed that discounting future losses and gains occurs asymmetrically in the brain (Xu et al., 2009). Lateral prefrontal and posterior parietal areas were activated more strongly in the loss condition. Moreover, the insula, thalamus, and dorsal striatum were more highly activated during intertemporal choices involving losses. In addition, whereas the posterior cingulate cortex and medial prefrontal cortex were activated when the choices included immediate options, extra regions, including the anterior cingulate cortex, insula and superior frontal gyrus, were preferentially activated when the choices involved immediate losses. Taken together, these findings suggest that future losses are discounted less steeply than future gains. The results of the present study support this possibility.

The absence of any significant difference in reaction times between the three groups might be partly interpreted that we did not have any requirements on time, so that some participants might waste time intentionally or unintentionally. Also, the delay discounting rate *k* was not significant, possibly because the *k* reflects the degree to which the subjective value decreases over time. In other words, the *k* reflects the slope of the discounting curve. The results suggested that high users differ from low users across time and value magnitude, but the slopes of the discounting curve were similar. That might be the reason why the *k* is not significant.

The present study explored smartphone high users’ intertemporal choice using a behavioral paradigm that differed from previous studies on smartphone high users, which have mainly used questionnaires and focused on traits like personality and gender. Grecucci et al. (2014) tested 23 normal controls and 23 diagnosed pathological gamblers in a behavioral intertemporal choice task. Their results showed that gamblers scored higher on impulsivity questionnaires, and selected a higher percentage of impatient choices when compared to normal controls. Their study also analyzed the two dimensions of time and money separately, and they found pathological gamblers showed more significant differences in different ΔAmount and ΔTime. The similar results in the present study suggested that smartphone high users have some of the same behavior patterns as other addicts. Indeed, some studies have called smartphone overuse “smartphone addiction” (Mok et al., 2014; Park and Lee, 2014; Bian and Leung, 2015; van Deursen et al., 2015).

Using the present methodology, we have revealed that there is a relationship between smartphone overuse and decision control, such that smartphone overuse is associated with a higher possibility of irrational decisions. What’s more, the present results suggest that smartphone use can exert an influence on intertemporal choices, even the frequency of usage is not particularly high, thus suggesting that smartphones might changing our beliefs in decision-making unwittingly. Considering the effects uncovered in the present study and those already described, perhaps it is time that we pay more attention to the problem of smartphone high use.

In sum, the present study revealed impaired decision control functioning in smartphone high users. High users tended to choose immediate reward and later penalty compared to low users, showing a characteristic of irrational decision-making. In addition, smartphone high users had different decision making strategies compared to low users for both dimensions of money and time. In addition, we provide an approximate range for how to define smartphone high users.

Some limitations must be acknowledged. The definition of the three groups was imprecise as we chose the upper 33.33%, the middle 33.33%, and the lower 33.33% of the SPAI score in order to classify high users group, medium users group, and low users group, respectively. And the samples of our study are all students, that might cause the sample not representative enough, so we will consider to use broader samples. Also, it would be better to set smartphone usage as a continuous variable, but our sample was too small to conduct a regression analysis. Hence future studies should rely on larger samples. So future studies could rely on larger sample. In addition, the present study could not reveal a causality between smartphone overuse and irrational decision making. Future studies could use other smartphone scales with a norm score to define high users more explicitly. To explore whether there is withdrawal syndrome like more established addictions, it could be beneficial to stop participants from using the smartphone. Another limitation relies on the fact that the intertemporal choice task we used was quite different from the titration method used by other authors in the past (Rodzon et al., 2011). The present paradigm presents the choices by random, which might make it less precise than titration method. Thus future studies could use some more subtle experimental manipulations. Finally, we should mention that the intertemporal choice task actually investigated preferences of participants when making choices in the lab rather than in real life. That is, someone choosing the LL in a lab-based intertemporal choice task, might not actually be willing to wait for an LL in reality. Future studies could use some form of delay-of-gratification tasks to further explore the impulsivity of smartphone high users.

## Author Contributions

ZT: completed the whole experiment, did the statistics, and wrote the paper. CQ: guide the whole research. HZ: take a part in the experiment design, and edited the paper. AY: did some of the literature review, and did some of the statistics.

## Conflict of Interest Statement

The authors declare that the research was conducted in the absence of any commercial or financial relationships that could be construed as a potential conflict of interest.
