# Supplementary material for: Time Is Money: The Decision Making of Smartphone High Users in Gain and Loss Intertemporal Choice
Source: Front Psychol. 2017 Mar 10;8:363. doi: 10.3389/fpsyg.2017.00363 (PMC5344929; doi:10.3389/fpsyg.2017.00363)
Supplement: Supplementary file 1 [file Data_Sheet_1.PDF]

# Supporting Information

## 1. BIS Score As A Covariate

### 1.1 Reaction times

We conducted a one-way ANOVA on reaction times (RTs, ms) using smartphone usage (overusers / slight overuses / underusers) as the independent variable, and the BIS score as a covariate. The difference between the three groups was not significant (overusers: $M = 2733.91$, $SD = 1205.42$; slight overusers: $M = 2656.40$, $SD = 1209.39$; underusers: $M = 2231.69$, $SD = 1018.35$; $F(2, 121) = 1.10$, $p = 0.335$, $\eta_p^2 = 0.018$, *power* = 0.240). And the main effect of BIS score was not significant, $F(2, 121) = 3.20$, $p = 0.076$, $\eta_p^2 = 0.026$, *power* = 0.427. These results indicated that trait impulsivity did not have an effect on the reaction time in intertemporal choice task.

### 1.2 Percentage of SS choices

To examine the differences in choices, we compared the percentage of SS option choices between the three groups, using the BIS score as a covariate. The results showed that the main effect of BIS score was not significant, $F(2, 121) = 0.022$, $p = 0.881$, $\eta_p^2 < 0.001$, *power* = 0.053. In the gain condition, there was a significant difference between the three groups, $F(2, 121) = 5.28$, $p = 0.006$, $\eta_p^2 = 0.080$, *power* = 0.827. Overusers selected 50.89% of SS in average ($SD = 0.18$), slight overusers selected 55.45% of SS in average ($SD = 0.16$), whereas underusers selected only 40.71% ($SD = 0.21$). Multiple comparisons suggested that the difference between underusers and slight overusers ($p = 0.002$) was significant, and the difference between underusers and overusers ($p = 0.055$) was marginal significant. In the loss condition, the difference between the three groups was not significant, $F(2, 121) = 2.23$, $p = 0.112$, $\eta_p^2 = 0.036$, *power* = 0.448. Overusers selected 74.83% of SS in average ($SD = 0.20$), slight overusers selected 79.06% of SS in average ($SD = 0.22$), whereas underusers selected 86.26% ($SD = 0.19$). However, multiple comparisons suggested that the difference between underusers and overusers ($p = 0.038$) was significant.

We also calculated the ΔAmount and the ΔTime. A mixed ANOVA was conducted using percentage of SS choices as the dependent variable, ΔAmount ($\Delta \leq 20$, $30 \leq \Delta \leq 60$, $\Delta \geq 70$) and valence (gain vs. loss) as within-subjects factors, group (overusers / slight overusers / underusers) as the between-subjects factor, and the BIS score as a covariate. The results showed that the main effect of BIS score was not significant, $F(2, 121) = 0.010$, $p = 0.919$, $\eta_p^2 < 0.001$, *power* = 0.053. The main effect of group was not significant ($F(2, 121) = 1.57$, $p = 0.211$, $\eta_p^2 = 0.025$, *power* = 0.329). The main effect of valence was significant ($F(1, 242) = 6.28$, $p = 0.014$, $\eta_p^2 = 0.049$, *power* = 0.701). Participants chose a significantly higher percentage of SS options in the loss condition compared to the gain condition. The main effect of ΔAmount was not significant ($F$(2,

242) = 2.04, $p$ = 0.132, $\eta_p^2$ = 0.017, *power* = 0.418). But the interaction of the three independent variables was marginal significant ($F$(4, 244) = 2.34, $p$ = 0.056, $\eta_p^2$ = 0.037, *power* = 0.672). Multiple comparisons showed that in the loss condition, only when it is small ΔAmount, overusers chose significantly fewer SS options than underusers ($p$ = 0.012). In the gain condition, slight overusers chose significantly more SS options than underusers in all ΔAmount ($p$ = 0.033 for small ΔAmount, $p$ < 0.001 for medium ΔAmount, $p$ = 0.004 for large ΔAmount), and overusers chose significantly more SS options than underusers with a medium ($p$ = 0.028) ΔAmount. However, overusers and slight overusers did not differ in all ΔAmount (see Table S1 for details).

**Table S1. Percentage of SS choices between groups in different ΔAmount.**

| **ΔAmount** | | Overusers | | Slight overusers | | Underusers | | Difference | | | |
|---|---|---|---|---|---|---|---|---|---|---|---|
| | | *M* | *SD* | *M* | *SD* | *M* | *SD* | *F* | Sig. | $\eta_p^2$ | *power* |
| Gain | ≤ 20 yuan | 0.84 | 0.21 | 0.87 | 0.21 | 0.74 | 0.28 | 2.36 | .099 | .038 | .470 |
| | ≥ 21 yuan & ≤ 59 yuan | 0.60 | 0.27 | 0.66 | 0.21 | 0.44 | 0.31 | 5.81 | .004 | .088 | .863 |
| | ≥ 60 yuan | 0.18 | 0.16 | 0.25 | 0.16 | 0.14 | 0.16 | 4.43 | .014 | .068 | .752 |
| Loss | ≤ 20 yuan | 0.49 | 0.35 | 0.62 | 0.35 | 0.73 | 0.35 | 3.26 | .042 | .051 | .610 |
| | ≥ 21 yuan & ≤ 59 yuan | 0.74 | 0.28 | 0.75 | 0.28 | 0.84 | 0.26 | 1.21 | .301 | .020 | .261 |
| | ≥ 60 yuan | 0.93 | 0.11 | 0.92 | 0.16 | 0.96 | 0.09 | 0.81 | .448 | .013 | .186 |

Then we calculated ΔTime. First, a mixed ANOVA was conducted using the percentage of SS choices as the dependent variable, ΔTime (3, 6, 9, 12 months) and valence (gain vs. loss) as within-subjects factors, group (overusers / slight overusers / underusers) as the between-subjects factor, and the BIS score as a covariate. The main effect of BIS score was not significant ($F$(2, 121) = 0.03, $p$ = 0.870, $\eta_p^2$ < 0.001, *power* = 0.053), and the main effect of group was not significant ($F$(2, 121) = 1.70, $p$ = 0.186, $\eta_p^2$ = 0.027, *power* = 0.352), whereas the main effect of valence was significant ($F$(1, 363) = 8.24, $p$ = 0.005, $\eta_p^2$ = 0.064, *power* = 0.813). Participants chose a significantly higher percentage of SS options in the loss condition compared to the gain condition. The main effect of ΔTime was not significant ($F$(3, 366) = 0.416, $p$ = 0.658, $\eta_p^2$ = 0.003, *power* = 0.117). The interaction between the three factors was not significant ($F$(6, 366) = 1.224, $p$ = 0.293, $\eta_p^2$ = 0.020, *power* = 0.482). In order to explore the detailed effects at specific time points, we conducted multiple comparisons of the specific time points in loss and in gain condition respectively. In the loss condition, the overusers chose significantly less SS options than underusers when Δ = 12 ($p$ = 0.023), and marginal significant when Δ = 3 and Δ = 9 ($p$ = 0.066 for Δ = 3, $p$ = 0.076 for Δ = 9), slight overusers chose marginal significantly fewer SS options than underusers when Δ = 3 ($p$ = 0.051), but overusers and slight overusers did not differ in each ΔTime. In the gain condition, slight overusers chose significantly more SS options than underusers in all ΔTime ($p$ = 0.017 for Δ = 3, $p$ = 0.033 for Δ = 6, $p$ = 0.001 for Δ = 9, $p$ = 0.002 for Δ = 12), but overusers and slight overusers did not differ in each ΔTime, overusers and underusers also did not

differ in each ΔTime (see Table S2 for details).

**Table S2. Percentage of SS choices between groups in different ΔTime.**

| ΔTime | | Overusers | | Slight overusers | | Underusers | | Difference | | | |
|---|---|---|---|---|---|---|---|---|---|---|---|
| | | *M* | *SD* | *M* | *SD* | *M* | *SD* | *F* | Sig. | $\eta_p^2$ | *power* |
| Gain | 3 | 0.33 | 0.16 | 0.36 | 0.13 | 0.26 | 0.19 | 2.94 | .057 | .046 | .563 |
| | 6 | 0.42 | 0.20 | 0.45 | 0.19 | 0.34 | 0.21 | 2.39 | .096 | .038 | .470 |
| | 9 | 0.58 | 0.21 | 0.65 | 0.21 | 0.46 | 0.25 | 5.72 | .004 | .086 | .858 |
| | 12 | 0.66 | 0.22 | 0.74 | 0.24 | 0.55 | 0.28 | 6.07 | .008 | .076 | .805 |
| Loss | 3 | 0.85 | 0.16 | 0.85 | 0.20 | 0.93 | 0.12 | 2.39 | .096 | .038 | .475 |
| | 6 | 0.81 | 0.18 | 0.84 | 0.20 | 0.90 | 0.17 | 1.30 | .276 | .021 | .278 |
| | 9 | 0.71 | 0.23 | 0.77 | 0.24 | 0.83 | 0.24 | 1.60 | .206 | .026 | .333 |
| | 12 | 0.64 | 0.28 | 0.73 | 0.27 | 0.81 | 0.26 | 2.67 | .074 | .042 | .521 |

### 1.3 Delay discounting rate (*k*)

Finally we conducted a mixed ANOVA with the *k* value as the dependent variable, valence (gain vs. loss) as the within-subjects factor, group (overusers / slight overusers / underusers) as the between-subjects factor, and the BIS score as a covariate. The main effect of BIS score was not significant ($F(2, 121) = 1.76$, $p = 0.188$, $\eta_p^2 = 0.014$, *power* = 0.260), and the effect of group was found not to be significant ($F(2, 121) = 1.21$, $p = 0.301$, $\eta_p^2 = 0.020$, *power* = 0.261). However, the effect of valence was marginal significant ($F(1, 121) = 3.86$, $p = 0.052$, $\eta_p^2 = 0.031$, *power* = 0.496). Participants had a higher discounting rate in the loss condition than in the gain condition. The interaction was not significant ($F(2, 121) = 1.64$, $p = 0.199$, $\eta_p^2 = 0.026$, *power* = 0.340).

### 1.4 Conclusion

These results revealed that in general, the trait impulsivity did not have a significant effect on intertemporal choice, but it could influence our choice at some specific time point or value magnitude. But our behavior in intertemporal choice task was mostly influenced by smartphone overuse.

## 2. The combination of different ΔAmount and different delay categories

In order to explore whether the combination of the different ΔTime and ΔAmount is proper, we test the differences between each ΔAmount and delay categories.

### 2.1 The test between different ΔAmount

An ANOVA was conducted using the percentage of SS choices as the dependent variable, ΔAmount (from 10 to 90) and valence (gain vs. loss) as within-subjects factors. The main effect of valence was significant ($F(1, 992) = 130.21$, $p < 0.001$, $\eta_p^2 = 0.512$,

*power* = 1.000), participants chose a significantly higher percentage of SS options in the loss condition compared to the gain condition. The main effect of ΔAmount was significant ($F(8, 992) = 55.60$, $p < 0.001$, $\eta_p^2 = 0.310$, *power* = 1.000), with larger ΔAmount, participants were less tend to choose SS options. The interaction between the two factors was significant ($F(8, 992) = 313.60$, $p < 0.001$, $\eta_p^2 = 0.717$, *power* = 1.000). Multiple comparisons revealed that in gain condition, the difference between any of two ΔAmount was significant, the larger ΔAmount is, the less SS options to choose; in loss condition, the difference between any of two ΔAmount was significant except Δ = 80 and Δ = 90 ($p = 0.410$), the larger ΔAmount is, the more SS options to choose (see Figure S1).

**Figure S1. Percentage of SS choices between gain and loss in different ΔAmount.**

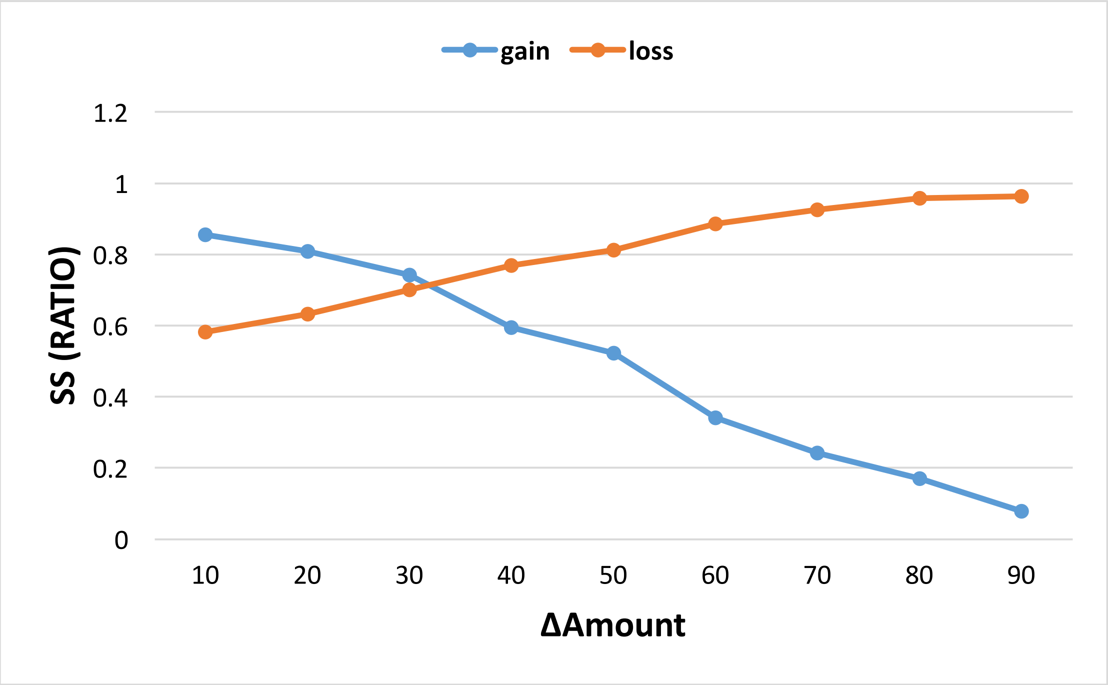


Since the differences were significant between almost all ΔAmount, and the percentage of SS choices with the ΔAmount increased steadily in loss condition and deceased steadily in gain condition. Thus we need to combine these ΔAmount into several levels to simplify the results. In the present study, we define the different ΔAmount levels as Grecucci et al (2014).

## 2.2 The test between different delay categories

In varying the waiting time, it is possible that the two pairs of each ΔTime were not equivalent, because some SS options were immediate and some were delayed. So we need to test the effect of different delay categories.

First we conducted ANOVAs between each two pairs for the same ΔTime using the percentage of SS choices as the dependent variable, different delay categories and

valence (gain vs. loss) as within-subjects factors. When ΔTime = 3, the main effect of valence was significant ($F(1, 124) = 571.55$, $p < 0.001$, $\eta_p^2 = 0.822$, *power* = 1.000), participants chose a significantly higher percentage of SS options in the loss condition compared to the gain condition. The main effect of delay category was significant ($F(1, 124) = 8.08$, $p = 0.005$, $\eta_p^2 = 0.061$, *power* = 0.805), participants choose significantly more SS options in "3 months versus 6 months" than "9 months versus 6 months". The interaction between the two factors was significant ($F(1, 124) = 58.84$, $p < 0.001$, $\eta_p^2 = 0.322$, *power* = 1.000). Multiple comparisons revealed that in gain condition, participants choose significantly more SS options in "3 months versus 6 months" than "9 months versus 6 months" but contrary in loss condition (both $p < 0.001$).

When ΔTime = 6, the main effect of valence was significant ($F(1, 124) = 278.30$, $p < 0.001$, $\eta_p^2 = 0.692$, *power* = 1.000), participants chose a significantly higher percentage of SS options in the loss condition compared to the gain condition. The main effect of delay category was significant ($F(1, 124) = 6.29$, $p = 0.013$, $\eta_p^2 = 0.048$, *power* = 0.702), participants choose significantly more SS options in "9 months versus 3 months" than "6 months versus 12 months". The interaction between the two factors was significant ($F(1, 124) = 16.98$, $p < 0.001$, $\eta_p^2 = 0.120$, *power* = 0.983). Multiple comparisons revealed that in gain condition, participants choose significantly more SS options in "9 months versus 3 months" than "6 months versus 12 months" ($p < 0.001$) but there was no significant difference in loss condition.

When ΔTime = 9, the main effect of valence was significant ($F(1, 124) = 37.40$, $p < 0.001$, $\eta_p^2 = 0.232$, *power* = 1.000), participants chose a significantly higher percentage of SS options in the loss condition compared to the gain condition. The main effect of delay category was not significant ($F(1, 124) = 0.10$, $p = 0.759$, $\eta_p^2 = 0.001$, *power* = 0.061). The interaction between the two factors was significant ($F(1, 124) = 69.63$, $p < 0.001$, $\eta_p^2 = 0.360$, *power* = 1.000). Multiple comparisons revealed that in gain condition, participants choose significantly more SS options in "today versus 9 months" than "12 months versus 3 months" ($p < 0.001$) but contrary in loss condition (both $p < 0.001$).

Then we defined a new variable: the waiting time of SS options. To explore how the waiting time of SS options and ΔTime influence the choice of individuals, and whether the two factors will influence each other, we conducted a MANOVA using the two factors as fixed factors, the percentage of SS options in gain and loss conditions as the dependent variable. The results suggested that in gain condition, the main effect of ΔTime was significant ($F(2,744) = 18.48$, $p < 0.001$, $\eta_p^2 = 0.047$, *power* = 1.000), with lager ΔTime, participants tend to choose more SS options. The main effect of SS waiting time was significant ($F(2,744) = 16.14$, $p < 0.001$, $\eta_p^2 = 0.042$, *power* = 1.000), the less SS waiting time is, the higher percentage of SS options be chosen. In loss condition, the main effect of ΔTime was not significant ($F(2,744) = 1.09$, $p = 0.338$, $\eta_p^2 = 0.003$, *power* = 0.241). The main effect of SS waiting time was significant ($F(2,744) = 10.56$, $p < 0.001$, $\eta_p^2 = 0.028$, *power* = 0.989), the more SS waiting time is, the higher

percentage of SS options be chosen.

But an important thing is that, both in gain and loss conditions, the interaction of the two factors was not significant (in gain condition: $F(2,744) = 0.76$, $p = 0.385$, $\eta_p^2 = 0.001$, *power* = 0.140; in loss condition: $F(2,744) = 0.64$, $p = 0.426$, $\eta_p^2 = 0.001$, *power* = 0.125). These results showed that the ΔTime and the different waiting time of SS options indeed have an effect on individuals' choices, but the two factors were independent. In the present study, we focus on the effect of ΔTime but not the different waiting time of SS options. Thus we only contained ΔTime in our results to make it more clearly.

**Reference**

Grecucci, A., Giorgetta, C., Rattin, A., Guerreschi, C., Sanfey, A. G., & Bonini, N. (2014). Time Devours Things: How Impulsivity and Time Affect Temporal Decisions in Pathological Gamblers. *PloS one, 9*(10), e109197.
